# Acupuncture for Tourette Syndrome: A Systematic Review

**DOI:** 10.1155/2016/1834646

**Published:** 2016-09-20

**Authors:** Jinna Yu, Yongming Ye, Jun Liu, Yang Wang, Weina Peng, Zhishun Liu

**Affiliations:** Acupuncture Department, Guang'anmen Hospital, China Academy of Chinese Medical Sciences, No. 5, Beixiange Street, Xicheng District, Beijing 100053, China

## Abstract

Tourette syndrome (TS) is a neuropsychiatric disorder that affects both children and adults. We searched for randomised controlled trials (RCTs) using acupuncture to treat TS written in English or Chinese without restrictions on publication status. Study selection, data extraction, and assessment of study quality were conducted independently by two reviewers. Meta-analyses were performed using Review Manager (RevMan) 5.3 software from the Cochrane Collaboration. Data were combined with the fixed-effect model based on a heterogeneity test. Results were presented as risk ratios for dichotomous data and mean differences (MDs) for continuous data. This review included 7 RCTs with a total of 564 participants. The combined results showed that acupuncture may have better short-term effect than Western medicine for TS and that acupuncture may be an effective adjuvant therapy in improving the effect of Western medicine on TS, but the evidence is limited because of existing biases. Rigorous high-quality RCTs are needed to verify these findings.

## 1. Background

Tourette syndrome (TS; also called Tourette's syndrome, Tourette's disorder, or Gilles de la Tourette syndrome) is an inherited neuropsychiatric disorder characterised by multiple physical (motor) tics and at least one vocal (phonic) tic present for more than 1 year [1]. These tics are characterised as sudden, rapid, recurrent, nonrhythmic motor movements or vocalisations usually appearing in bouts that can wax and wane in frequency, intensity, and diversity [2]. The population prevalence estimate of TS in children is 0.3–0.9% [3], and TS is 2–4 times more common in boys than girls [4]. Most cases are associated with other comorbidities such as obsessive compulsive disorder, attention deficit hyperactivity disorder, schizophrenia, and mental impairment. TS and these combined conditions cause impairment in people living with TS [5].

The European Society for the Study of Tourette Syndrome (ESSTS) guidelines developed in 2011 recommend that TS could be treated by medications including alpha-adrenergic agonists, typical neuroleptics, atypical neuroleptics, and benzamides [6, 7]. Medications cannot eradicate tics completely, and they can cause adverse reactions such as extrapyramidal symptoms and weight gain with atypical neuroleptics and drowsiness and extrapyramidal motoric adverse reactions with typical antipsychotics [7–10]. Therefore, there is a need for other effective and safe treatment methods for TS.

In recent years, complementary and alternative medicine (CAM) has been used to treat TS increasingly, including prayer, vitamins, massage, dietary supplements, chiropractic manipulations, meditation, diet alterations, yoga, hypnosis, homeopathy, EEG biofeedback, and acupuncture [11]. 73.6%–87.8% of TS patients selected CAM [11, 12], and 56% of patients reported improvement after CAM treatment [11].

Acupuncture is a branch of traditional Chinese medicine that dates back thousands of years [13]. It is defined as fine needles piercing into the acupoints based on traditional Chinese medicine theory. It has been reported that acupuncture can regulate the abnormal brain function of patients with tic disorders [14] and can alleviate tic symptoms [15–17]. To date, there is only one published Chinese systematic review on acupuncture for TS [18]; five RCTs and one quasi-RCT published between 2001 and 2009 were included and analysed, and all included studies were published in Chinese. This review indicated that acupuncture had a better effect than conventional Western medicine on response rate according to a Chinese criterion [19] but no difference in Yale Global Tic Severity Scale (YGTSS) evaluation; however, the studies included in the previous review were not all RCTs, and there was a high risk of bias in some studies. Nearly 20 RCTs regarding acupuncture and TS have been published since the last review [16, 20–35]. With a more comprehensive search strategy and more included databases, the present systematic review aims to update and evaluate whether acupuncture is effective and safe for alleviating the symptoms of TS.

## 2. Methods

### 2.1. Study Criteria

#### 2.1.1. Types of Studies

RCTs written in English or Chinese were included, without restriction of publication status. We excluded quasi-randomised studies such as those with evidence of inadequate sequence generation, for example, alternate days or patient numbers.

#### 2.1.2. Types of Participants

Studies including patients of any age who met the medically defined diagnostic criteria for TS were eligible for inclusion. Diagnostic criteria may have been from the Diagnostic and Statistical Manual, third edition (DSM-III), fourth edition (DSM-IV), or text revision of the fourth edition (DSM-IV-TR); the International Classification of Diseases, tenth version (ICD-10); the Chinese Classification of Mental Disorders, second revision version (CCMD-2R) and third version (CCMD-3); or other clearly defined diagnostic criteria. Studies including patients whose tics were transient tic disorders, chronic motor or vocal tic disorders, or tic disorders caused by medication were excluded.

#### 2.1.3. Types of Interventions

Acceptable interventions included manual acupuncture, electroacupuncture, scalp acupuncture, auricular acupuncture, warm needling, plum-blossom needling, or intradermal needling. Acupoint injections, acupoint catgut embedding, and laser acupuncture were excluded.

Comparison interventions included no intervention/waiting list control, placebo/sham acupuncture, or active Western medicine. Studies evaluating acupuncture combined with Western medicine compared with the same Western medicine alone were included.

Studies that compared different acupoints, compared two different forms of acupuncture, compared acupuncture with a Chinese patent or decoction, or compared acupuncture plus Chinese medicine with Chinese medicine alone were excluded.

#### 2.1.4. Types of Outcome Assessments

*(i) Primary Outcomes*. The most widely used checklists on tic characteristics and severity include the YGTSS, the Shapiro Tourette Syndrome Severity Scale (STSSS), and the Hopkins motor and vocal tic scale. Different scales focus on different aspects. The YGTSS includes 30 items including 18 categories of motor and vocal tics, self-injurious behaviour, and anger control problems and also a severity rating scale. The STSSS consists of five items including the noticeability to others and interference of daily life due to tics [2]. In this review, only the YGTSS was used in all included studies.

*(ii) Secondary Outcomes*

*(1) Response Rate*. Studies that reported the response rates as dichotomous measures (effective or ineffective) were considered. In some studies, patients with reduction rate of YGTSS less than 30% were regarded as “ineffective” and others as “effective.” These studies were included in this review. According to the criterion in China [19], some studies [30, 31, 39, 40] defined patients with no improvement in symptoms as “ineffective” and others as “effective,” but there was no quantified standard for improvement in this criteria, so we excluded these studies. 

*(2) Quality of Life (QOL)*. This was assessed by the health-related quality of life scale developed specifically for tic disorder patients [41], the Chinese Children and Adolescents' QOL Scale [42], and other QOL scales. None of the included studies used QOL as an outcome measure.

#### 2.1.5. Adverse Reactions

All adverse reactions and acupuncture-related adverse reactions were analysed.

### 2.2. Search Methods for the Identification of Studies

We searched the Cochrane Movement Disorders Group Specialised Register of Controlled Trials, the Cochrane Central Register of Controlled Trials, MEDLINE, EMBASE, PsycINFO, and Cumulative Index to Nursing and Allied Health Literature. We also searched four Chinese electronic databases: Chinese BioMedical Literature Database, China National Knowledge Infrastructure, VIP Database for Chinese Technical Periodicals, and Wanfang Digital database.

Other electronic sources searched for relevant trials included

1. trial registers for ongoing and registered trials: http://www.clinicaltrials.gov/, http://www.who.int/trialsearch/Default.aspx;
2. reference lists from relevant reviews and trials: the Cochrane Library Database of Abstracts of Reviews of Effects (reference lists from non-Cochrane reviews on similar topics);
3. conference abstracts on the Web of Knowledge (http://wokinfo.com/);
4. OpenGrey for unpublished literature from Europe (http://www.opengrey.eu/).

The date of the last search was March 28, 2016. The search strategy used is detailed in Table 1.

### 2.3. Data Collection and Analysis

#### 2.3.1. Selection of Studies

**Figure 1:**
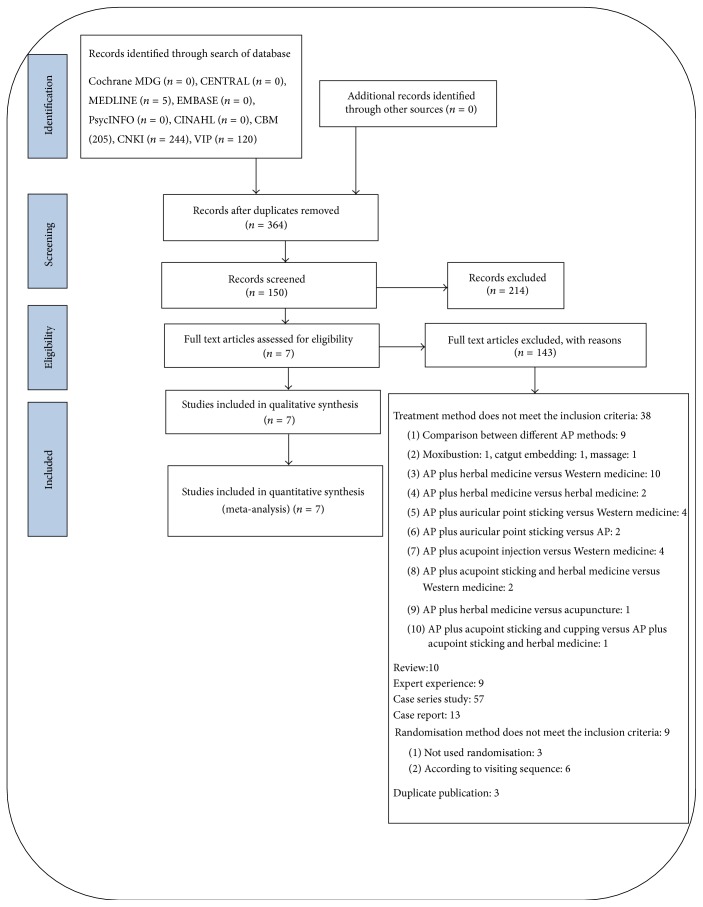
PRISMA flow chart. CENTRAL: the Cochrane Central Register of Controlled Trials; CINAHL: Cumulative Index to Nursing and Allied Health Literature; CBM: Chinese Biomedical Literature Database; CNKI: China National Knowledge Infrastructure Database; VIP: a database for Chinese Technical Periodicals.

After an initial screening of titles and abstracts retrieved by the search, the full text of all potentially eligible studies was retrieved. Two review authors (YJ and LJ) independently examined these full text articles for compliance with the inclusion criteria and selected studies eligible for inclusion in the review. Disagreements about study eligibility were resolved by discussion or by a third review author (LZ). The selection process was documented in a “PRISMA” flow chart (Figure 1).

#### 2.3.2. Data Extraction and Management

YJ and LJ independently extracted data from included studies using a data extraction form. Any disagreements were resolved by discussion or by a third review author (LZ). The extraction form included the following sections:

1. General study information.
2. Methods, including randomisation, blinding, and trial design.
3. Participants, including country, site, recruitment, inclusion, and exclusion.
4. Interventions, including methods of each group, duration, follow-up data, and withdrawals.
5. Outcomes, including primary and secondary outcomes.
6. Risk of bias, including the method of randomisation, allocation concealment, trial completion, and other bias.

We attempted to contact the original authors by telephone to obtain any incomplete data.

#### 2.3.3. Assessment of the Risk of Bias in Included Studies

Two reviewers independently assessed the included studies for risk of bias using the Cochrane risk of bias assessment tool (Cochrane Handbook, version 5.1.0; March 2011). Reviewers assessed selection (random sequence generation and allocation concealment), performance (blinding of participants and personnel), detection (blinding of outcome assessors), attrition (incomplete outcome data), reporting (selective reporting), and other sources of bias. The assessments were categorised into three levels of bias: low risk, high risk, and unclear risk. Disagreements were resolved by discussion or by a third review author (LZ).

#### 2.3.4. Data Synthesis and Analysis

RevMan 5.3 software was used to conduct the statistical analysis. Studies were classified and combined in the analysis according to different comparisons. To assess the heterogeneity across the studies, we searched for overlapping confidence intervals (CI) in forest plots and used the *χ* ^2^ test for statistical heterogeneity and the value of the *I* ^2^ statistic (*I* ^2^ > 50% showed the existence of heterogeneity). There was no significant heterogeneity in each group, so meta-analyses were all conducted with fixed-effect models in this review. For dichotomous data, we combined the risk ratios (RRs) of each study and calculated the 95% CI with the fixed-effect model. For continuous data, we combined the mean differences (MDs) of each study and calculated the 95% CI according to the outcome measurement. Publication bias was not explored via a funnel-plot analysis as the number of included studies in each group was less than 10.

## 3. Results

### 3.1. Characteristics of Included Studies and Literature Search Findings

The initial search identified 760 studies, of which 150 full text articles were screened, and a final total of 7 RCTs were included in this review [29, 32–34, 36–38]. All of the 7 RCTs were reported in Chinese and conducted in China.

Five studies compared acupuncture with Western medicine. Among them, two compared acupuncture with haloperidol [29, 36], one compared acupuncture with risperidone [32], and one compared acupuncture with tiapride [33]. Another one [34] compared acupuncture plus psychological behaviour therapy with haloperidol plus psychological behaviour therapy, which was regarded as comparison between acupuncture and haloperidol.

Two studies estimated the effect of acupuncture as an adjuvant therapy of Western medicine. One compared acupuncture plus haloperidol versus haloperidol alone [37], and the other one compared acupuncture plus haloperidol and psychological therapy versus haloperidol and psychological therapy [38]. The second study [38] was regarded as “comparing acupuncture plus haloperidol versus haloperidol alone” and was meta-analysed with the first study mentioned above [37].

The 7 RCTs included a total of 564 patients, with sample size ranging from 45 to 120. The age of the participants ranged from 2 to 21 years. All of the participants met the diagnostic criteria. The treatment duration ranged from 20 days to 3 months. In these studies, the acupoints that were commonly used were Baihui (GV20) (6/7), Fengchi (GB20) (5/7), Taiyang (EX-HN5) (5/7), Hegu (LI4) (4/7), Shenmen (HT7) (4/7), and Yintang (EX-HN3) (3/7). The most frequently used treatment methods were manual acupuncture (3/7) and electroacupuncture (3/7). The commonly used treatment frequency was once a day (6/7). The characteristics of included studies are listed in Table 2.

### 3.2. Risks of Bias in Included Studies

All of the included RCTs mentioned randomisation; six RCTs used a random number table, the other RCT did not report the method of randomisation and we were unable to contact the authors [32]. The details of allocation concealment and blinding of outcome assessors were unclear in all studies. Blinding of participants was not done in any of the studies because of the characteristics of acupuncture. One RCT reported incomplete outcome data but did not conduct an intention to treat (ITT) analysis [38]. Neither RCT reported follow-up. The risks of bias assessments are presented in Table 3.

### 3.3. Effects of Intervention

The 7 included RCTs were divided into two parts to conduct the meta-analysis according to different types of comparisons.

### 3.4. Acupuncture versus Western Medicine

#### 3.4.1. Yale Global Tic Severity Scale

**Figure 2:**
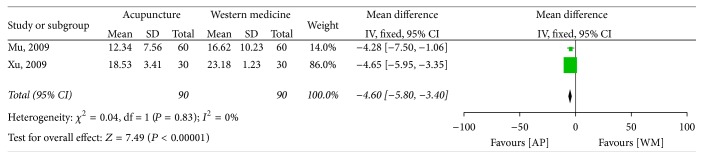
Forest plot of the effect of acupuncture versus Western medicine on the Yale Global Tic Severity Scale. AP: acupuncture; WM: Western medicine.

In the five studies which compared acupuncture with Western medicine, only two RCTs used the YGTSS as an outcome measure, and they were pooled together [32, 34]. The MD was −4.60 (95% CI −5.80 to −3.40) using the fixed model, and there was a statistically significant difference between the effects of acupuncture and Western medicine (Figure 2).

#### 3.4.2. Response Rate

**Figure 3:**
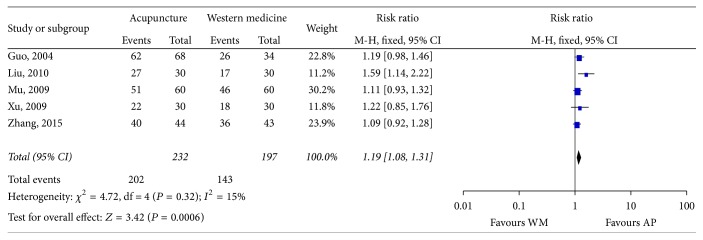
Forest plot of the effect of acupuncture versus Western medicine on response rate. AP: acupuncture; WM: Western medicine.

Five studies [29, 32–34, 36] reported response rate here with the RR as 1.19 (95% CI 1.08 to 1.31), which showed that there was a statistically significant difference between the effects of acupuncture and Western medicine (Figure 3).

### 3.5. Acupuncture plus Western Medicine versus Western Medicine Alone

Two studies were included in this group. In one study [38], 60 participants were initially randomised into the acupuncture group, but only 56 finished the treatment. We did an ITT analysis on the “response rate” as a dichotomous outcome but not for the YGTSS because of a lack of original data for the missing four participants.

#### 3.5.1. Yale Global Tic Severity Scale

**Figure 4:**
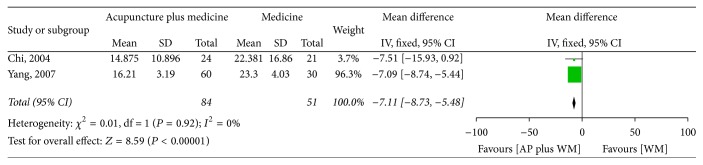
Forest plot of the effect of acupuncture plus Western medicine versus Western medicine alone on the Yale Global Tic Severity Scale. AP: acupuncture; WM: Western medicine.

Two RCTs were pooled together [37, 38]. The MD was −7.11 (95% CI −8.73 to −5.48) using the fixed model, and there was a statistically significant difference between the effects of acupuncture plus Western medicine and Western medicine alone (Figure 4).

#### 3.5.2. Response Rate

**Figure 5:**
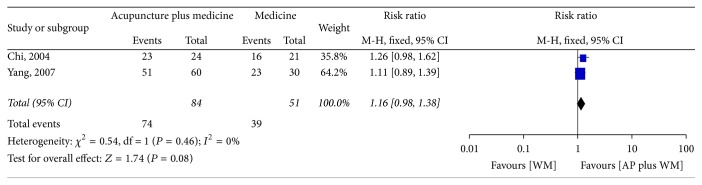
Forest plot of the effect of acupuncture plus Western medicine versus Western medicine alone on response rate. AP: acupuncture; WM: Western medicine.

In this group, two studies were pooled together [37, 38]. The RR was 1.16 (95% CI 0.98 to 1.38) using the fixed model, from which we could not make a conclusion whether acupuncture plus Western medicine had better effect than Western medicine alone (Figure 5).

### 3.6. Follow-Up

No study reported follow-up after treatment.

### 3.7. Safety

One RCT reported no adverse reactions in the acupuncture group [29], and another RCT reported no adverse reactions in the 56 participants in the acupuncture plus haloperidol and psychotherapy group [38]. The other five included RCTs did not report whether there were adverse reactions in participants.

## 4. Discussion

### 4.1. Summary of Main Findings

Despite an extensive literature search, only 7 studies involving a total of 564 patients were eligible for our review. The result of meta-analysis indicated that, compared with Western medicine, acupuncture seemed to be more effective in improving the YGTSS (MD −4.60, 95% CI −5.80 to −3.40) and in response rate 1.19 (95% CI 1.08 to 1.31). Data from two studies showed that acupuncture as an adjuvant therapy enhanced the effect of Western medicine (MD −7.11, 95% CI −8.74 to −5.47) in improving the YGTSS, while it is inconclusive whether acupuncture could improve the response rate of Western medicine (RR 1.16, 95% CI 0.98 to 1.38). There was no report on follow-up of acupuncture on TS. Acupuncture seems to be associated with no adverse reactions, but the evidence is limited.

### 4.2. Applicability of Evidence

There were methodological limitations of included studies as no study reported the allocation concealment, which led to unclear selection bias. No study used participants and personnel blinding which led to high performance, and there was no report on assessment blinding which led to unclear detection bias. One study reported 4/90 (<5%) patients fell off and other studies reported that all patients finished the assessment. No protocol was published before outcome carried out, which led to unclear reporting bias in all studies. All studies were conducted in China, which made it unclear whether the conclusion of this review is fit for other races. Methodological weaknesses in the studies might have exaggerated the treatment effects of acupuncture.

Haloperidol and risperidone are both effective medicines recommended by the ESSTS. The effect of acupuncture may be better than these medicines, which may indicate that acupuncture may have clinical application value. However, we do not know clearly about the design of the included studies, such as superiority test and noninferiority test, and there is also a lack of sample size calculations in some studies. No study used placebo/sham acupuncture as a comparison, and therefore it is unclear whether acupuncture has a specific biological treatment effect.

### 4.3. Difference from Other Reviews

Six studies [36, 40, 43–46] published from 2001 to 2009 were included in the published Chinese review [18]. Among them, five [40, 43–46] were excluded and one [36] was included in our review. Of the excluded studies, one study was a quasi-randomised study that allocated treatment by patient number [43], two studies used false randomisation [44, 45], one study used incorrect outcome measure, and another study [46] was duplication of one study [39] that was included in our review. There are other differences between these two reviews explained as follows. First, one study included in the published Chinese review compared acupuncture plus point injection with medication [43], which makes it impossible to tell whether the treatment effect was produced by acupuncture or the injected medicine. In our review, acupuncture combined with other methods (e.g., acupressure, acupoint injection, and moxibustion) were all excluded. Second, the published Chinese review was published in Chinese; our review was written in English. Finally, the published Chinese review indicated that acupuncture had a better effect than conventional Western medicine on response rate but caused no difference in YGTSS evaluation. In our review, we found that acupuncture seemed to be more effective than Western medicine and seemed to be an effective method as an adjuvant therapy of Western medicine in YGTSS evaluation, and we also found that acupuncture may have a better effect than Western medicine in response rate, but the evidence is limited.

### 4.4. Mechanism and Limitations of Acupuncture on TS

Although acupuncture was widely used in TS treating in China, there is only one study [14] researched on the mechanism of acupuncture for TS by now. It reported acupuncture may regulate the abnormal brain function of patients with tic disorder using single photon emission computed tomography (SPECT). Acupuncture also has limitations in treating TS children. Maintaining a fixed position during conventional acupuncture is difficult for children, and the pain produced during conventional acupuncture treatment leads to poor compliance. Suitable acupuncture method is more feasible for TS children, such as plum-blossom needle therapy and acupoint embedding. Researches on mechanism of acupuncture for TS are also urgently needed.

### 4.5. Limitations of This Review

There are some limitations to this review. First, the size of included studies is small. Second, only Chinese and English databases were searched, so some relevant studies published in other languages might have been missed. Third, despite the difference among different acupuncture methods in this review (such as manual acupuncture, electroacupuncture, and scalp acupuncture), we still combined them together because of small number of studies of each acupuncture type. But this combination will make us unclear about the effect of each acupuncture type. Deeper subgroup analysis can be done when the number of studies is enough. Finally, some useful outcome measures have not been used in included studies. For example, “quality of life” is a good outcome measure that was not reported in any included study.

## 5. Conclusion

Based on the present findings, we cautiously suggest that acupuncture may have better short-term effect than Western medicine for TS in alleviating the symptoms and that acupuncture may be an effective adjuvant therapy in improving the effect of Western medicine on TS. However, the evidence is limited. There is a need for large-scale and well-designed RCTs of acupuncture for TS with rigorous methods of randomisation, blinding, and adequately concealed allocation, as well as validated outcome measures. All information including adverse effects should be reported in detail, according to both CONSORT [47] and CONSORT for acupuncture [48].

## Figures and Tables

**Table 1:** Search strategy used in electronic databases.

Number	Search items
1	Randomised controlled trial
2	Controlled clinical trial
3	Randomised
4	Randomized
5	Randomly
6	Placebo
7	Trial
8	1 or 2–7
9	Tourette Syndrome
10	Tourette Disorder
11	Gilles de la Tourette Syndrome
12	Tourette^*∗*^
13	Tic
14	9 or 10–13
15	Acupuncture therapy
16	Acupuncture
17	Body acupuncture
18	Scalp acupuncture
19	Auricular acupuncture
20	Electroacupuncture
21	Intradermal needling
22	Plum-blossom needle
23	Acupoints
24	Acu^*∗*^
25	15 or 16–24
26	8 and 14 and 25

Tourette^*∗*^: any words or phrases starting with “tourette”. Acu^*∗*^: any words or phrases starting with “acu”.

**Table 2:** Summary of characteristics of included studies.

Reference	Comparisons	Drug dose	Age (years)	Duration of illness (years)	YGTSS	Outcomes	Duration of intervention	Duration of follow-up
Guo et al., 2004 [36]	Electroacupuncture (*n* = 68)	1.5–8 mg/d	NR	1 to 8 in two groups	NR	Response rate	30 days (30 sessions)	No follow-up
	Haloperidol (*n* = 34)							

Liu et al., 2010 [30]	Electroacupuncture (*n* = 30)	1.5–8 mg/d	NR	1 to 5	NR	Response rate	20 days (20 sessions)	No follow-up
	Haloperidol (*n* = 30)			1 to 5				

Xu and Zhu, 2009 [33]	Scalp acupuncture (*n* = 30)	0.25–1 mg/d	6 to 18	1 to 7	30.07 ± 2.76	YGTSS, response rate	3 months (39 sessions)	No follow-up
	Risperidone (*n* = 30)		4 to 17	1 to 8	35.03 ± 3.46			

Zhang et al., 2015 [34]	Manual acupuncture (*n* = 44)	150–450 mg/d	2 to 15 in two groups	0.5 to 3.2 in two groups	NR	Response rate	3 months (60 sessions)	No follow-up
	Tiapride (*n* = 43)							

Mu et al., 2009 [35]	Electroacupuncture plus psychological behaviour therapy (*n* = 60)	From 1 mg/d	10.24 ± 3.41	2.15 ± 1.07	33.61 ± 5.76	YGTSS, response rate	6 weeks (42 sessions)	No follow-up
	Haloperidol plus psychological behaviour therapy (*n* = 60)		9.53 ± 2.75	2.48 ± 1.55	31.08 ± 6.54			

Chi and Sun, 2004 [37]	Manual acupuncture plus haloperidol (*n* = 24)	1.5–8 mg	2 to 21	1 to 9	39.875 ± 15.875	YGTSS, response rate	20 days (20 sessions)	No follow-up
	Haloperidol (*n* = 21)		2 to 21	1 to 9	40.190 ± 15.863			

Yang et al., 2007 [38]	Manual acupuncture plus haloperidol and psychotherapy (*n* = 60 initially; final *n* = 56)	From 1 mg/d	11.52 ± 2.96	NR	34.26 ± 5.88	YGTSS, response rate	40 days (40 sessions)	No follow-up
	Haloperidol and psychotherapy (*n* = 30)		12.03 ± 3.37		36.01 ± 6.73			

YGTSS: Yale Global Tic Severity Scale. NR: no reported.

**Table 3:** Risk of bias assessment of included studies.

Bias	Guo et al., 2004 [36]	Liu et al., 2010 [30]	Xu and Zhu, 2009 [33]	Zhang et al., 2015 [34]	Mu et al., 2009 [35]	Chi and Sun, 2004 [37]	Yang et al., 2007 [38]
Random sequence generation (selection bias)	Random number table (LR)	Random number table (LR)	NR (UR)	Random number table (LR)	Random number table (LR)	Random number table (LR)	Random number table (LR)

Allocation concealment (selection bias)	UR	UR	UR	UR	UR	UR	UR

Blinding of participants and personnel (performance bias)	HR	HR	HR	HR	HR	HR	HR

Blinding of outcome assessment (detection bias)	UR	UR	UR	UR	UR	UR	UR

Incomplete outcome data (attrition bias)	LR	LR	LR	LR	LR	LR	LR

Selective reporting (reporting bias)	UR	UR	UR	UR	UR	UR	UR

Other bias	LR	LR	LR	LR	LR	LR	LR

UR: unclear risk; LR: low risk; HR: high risk; NR: no reported.

## Abbreviations

CI: Confidence intervals
ESSTS: European Society for the Study of Tourette Syndrome
ITT: Intention to treat
MD: Mean difference
RCT: Randomised controlled trial
STSSS: Shapiro Tourette Syndrome Severity Scale
TS: Tourette syndrome
YGTSS: Yale Global Tic Severity Scale.
